# Bleomycin-Loaded pH-Sensitive Polymer–Lipid-Incorporated Liposomes for Cancer Chemotherapy

**DOI:** 10.3390/polym10010074

**Published:** 2018-01-15

**Authors:** Eiji Yuba, Tomohiro Osaki, Misato Ono, Shinjae Park, Atsushi Harada, Masamichi Yamashita, Kazuo Azuma, Takeshi Tsuka, Norihiko Ito, Tomohiro Imagawa, Yoshiharu Okamoto

**Affiliations:** 1 Department of Applied Chemistry, Graduate School of Engineering, Osaka Prefecture University, 1-1 Gakuen-cho, Naka-ku, Sakai, Osaka 599-8531, Japan; ma105114@edu.osakafu-u.ac.jp (S.P.); harada@chem.osakafu-u.ac.jp (A.H.); 2 Joint Department of Veterinary Clinical Medicine, Faculty of Agriculture, Tottori University, 4-101 Koyama-Minami, Tottori 680-8553, Japan; misatosu202@gmail.com (M.O.); yamashita@muses.tottori-u.ac.jp (M.Y.); kazu-azuma@muses.tottori-u.ac.jp (K.A.); tsuka@muses.tottori-u.ac.jp (T.T.); taromobile@me.com (N.I.); imagawat@muses.tottori-u.ac.jp (T.I.); yokamoto@muses.tottori-u.ac.jp (Y.O.)

**Keywords:** bleomycin, cancer, pH-sensitive liposome, polyglycidol, polymer–lipid, poly(ethylene glycol)

## Abstract

Cancer chemotherapeutic systems with high antitumor effects and less adverse effects are eagerly desired. Here, a pH-sensitive delivery system for bleomycin (BLM) was developed using egg yolk phosphatidylcholine liposomes modified with poly(ethylene glycol)-lipid (PEG-PE) for long circulation in the bloodstream and 2-carboxycyclohexane-1-carboxylated polyglycidol-having distearoyl phosphatidylethanolamine (CHexPG-PE) for pH sensitization. The PEG-PE/CHexPG-PE-introduced liposomes showed content release responding to pH decrease and were taken up by tumor cells at a rate 2.5 times higher than that of liposomes without CHexPG-PE. BLM-loaded PEG-PE/CHexPG-PE-introduced liposomes exhibited comparable cytotoxicity with that of the free drug. Intravenous administration of these liposomes suppressed tumor growth more effectively in tumor-bearing mice than did the free drug and liposomes without CHexPG-PE. However, at a high dosage of BLM, these liposomes showed severe toxicity to the spleen, liver, and lungs, indicating the trapping of liposomes by mononuclear phagocyte systems, probably because of recognition of the carboxylates on the liposomes. An increase in PEG molecular weight on the liposome surface significantly decreased toxicity to the liver and spleen, although toxicity to the lungs remained. Further improvements such as the optimization of PEG density and lipid composition and the introduction of targeting ligands to the liposomes are required to increase therapeutic effects and to reduce adverse effects.

## 1. Introduction

Cancer chemotherapy is a standard therapy to treat malignant tumors. Anticancer drugs can kill cancer cells and suppress tumor growth, although systemic distribution of these drugs exerts adverse effects on normal cells and normal tissues. Therefore, the selective delivery of anticancer drugs to tumors and avoidance of drug distribution to normal cells would improve cancer therapeutic effects and improve patients’ quality of life during cancer treatments. For this purpose, drug delivery systems (DDSs), which control drug release from carriers, drug absorption to target tissues, and selective delivery of the drug to target cells, have been eagerly studied for some decades [1,2,3]. Drug carriers of around 100 nm in size are known to accumulate efficiently in tumor tissues because of the leaky blood vessel structure in tumor tissues and the lack of lymphatic systems compared with normal tissues. This phenomenon is known as the Enhanced Permeation and Retention effect, or EPR effect [4]. Long circulation in the blood can be provided by surface modification of nanocarriers with poly(ethylene glycol) (PEG), which suppresses the opsonization and recognition by mononuclear phagocyte systems (MPS) via its highly hydrated structure [3]. In fact, some PEG-modified liposomes encapsulating anticancer drugs such as doxorubicin (DOX) are clinically available for cancer treatments. Moreover, PEG-coated polymeric carriers have been studied extensively in clinical trials [5,6]. These carriers accumulate in tumor tissues via the EPR effect and release anticancer drugs at the tumor site after decomposition or cleavage of the polymer–drug linkage responding to phospholipase or protease in situ. However, recent reports from the literature have stated that the clinical therapeutic effects of DOX-loaded liposomes are almost identical to those of free DOX [7]. This suggests that further improvements of clinically available liposomal DDS are required, such as active drug release responding to internal or external stimuli, selective association of carriers to tumor cells, and the control of the intracellular behavior of carriers.

The introduction of stimuli-responsive properties to DDS carriers is a useful strategy to achieve many requirements of DDS-based cancer chemotherapy [2,8]. Temperature-sensitive carriers have been studied to control drug release by external heating [9,10,11]. Other external stimuli such as magnetic fields and light irradiation have also been used to increase carrier accumulation to target sites and for local heating of tumors [12,13,14,15,16,17]. On the other hand, pH is a representative and well-used internal stimulus for DDS [18]. Tumor sites and inflammatory sites possess lower pH than the normal physiological pH. Additionally, the weakly acidic pH at intracellular acidic organelles (endosomes/lysosomes) is useful for triggering drug release from carriers. In fact, pH-sensitive liposomes have been studied eagerly as drug carriers to release drugs at tumor sites or in endosomes/lysosomes [19,20,21,22,23,24,25,26].

Typical pH-sensitive liposomes are prepared using cholesteryl hemisuccinate and dioleoylphosphatidylethanolamine, which are, respectively, a pH-responsive amphiphile and a non-bilayer-forming lipid [27]. At neutral pH, the carboxylates in cholesteryl hemisuccinate increase the hydration of the particle surface, leading to bilayer formation. In contrast, these liposomes change to a hexagonal II phase after protonation of the carboxyl groups and release their contents immediately [27]. Also, pH-sensitive liposomes have been prepared using bilayer-forming amphiphiles with chargeable head groups [28]. After protonation of the chargeable groups at weakly acidic pH, such as tertiary amines, the hydrophobic–hydrophilic balance of amphiphile changed from a bilayer to a micelle-forming property, which achieved complete drug release from carriers within a minute [28]. These amphiphile-based pH-sensitive liposomes show quite sharp pH-responsiveness because of the quick phase transition of amphiphiles, whereas the responsive pH is fixed by the intrinsic property of each amphiphile.

The modification of pH-responsive polymers onto liposomes is another strategy for preparing pH-sensitive liposomes. Polycarboxylates, which undergo coil-to-globule transition after protonation of the carboxyl groups, are typical pH-responsive polymers used for the pH-sensitizing of liposomes. Vinyl polymers, polyglycidols, and biodegradable polysaccharide-based pH-responsive polymers have been developed to induce content release at weakly acidic pH [29,30,31,32,33,34]. An important advantage of pH-responsive polymers over pH-responsive lipids is their flexibility of polymer design, which enables control of the desired response-inducing pH by change of the polymer structure. However, control of polymer modification amounts on liposomes is difficult because polymer modification is usually attempted via hydrophobic interaction with the lipid membrane and randomly introduced alkyl chains in polymers.

Already, pH-responsive polymer–lipids with phospholipid anchors have been developed to take advantage of both lipid-based and polymer-based pH-sensitive liposomes [35]. Carboxylated polyglycidols have been connected to distearoyl phosphatidylethanolamine [35]. These polymer–lipid-incorporated egg yolk phosphatidylcholine (EYPC) liposomes showed content release with pH decrease and delivered their contents to the interior of cells [35]. These liposomes were also applied to antigenic protein delivery to immune cells for the induction of antigen-specific immune response [35]. A spacer group between the carboxyl group and ester group in the polyglycidol moiety strongly affects their pH-sensitivity and delivery performance. In particular, 2-carboxycyclohexane-1-carboxylated polyglycidol-having lipid (CHexPG-PE, Figure S1a) exhibited sharp pH-responsiveness after incorporation into EYPC liposomes, along with strong intracellular delivery performance [35]. CHexPG-PE-incorporated liposomes were further applied to human cancer-derived antigenic peptide delivery for the promotion of cross-presentation and antigen-specific cellular immunity [36,37]. Consequently, CHexPG-PE-incorporated liposomes achieved efficient intracellular delivery of low molecular fluorescent dye, protein, and peptides, although no report describes anticancer drug delivery using these liposomes.

For this study, bleomycin (BLM, Figure S1b) was used as a chemotherapeutic agent. BLM, a water-soluble antibiotic derived from *Streptornyces verticillus*, shows antitumor effects for cancers of several types (e.g., testicular cancers, lymphomas, and germinal cell tumors in children) by DNA cleavage via production of reactive oxygen species after complexation with Fe ions inside cells [38,39,40]. BLM shows bone-marrow suppression to only a slight degree, which is a typical adverse effect of general anticancer drugs, but administration of BLM causes other adverse effects such as interstitial pneumonia, lung fibrosis, pyrexia, and emesis [38,39,40]. To improve the bioavailability of BLM and to decrease its adverse effects, BLM-conjugated gold nanoparticles, magnetic nanoparticles, nanogels, and liposomes have been reported [41,42,43,44,45,46,47]. Although folate-PEG-modified liposomes achieved targeted delivery to tumor cells via folate receptor-mediated endocytosis and tumor inhibition in vivo, BLM release behavior from liposomes was slow: 50% of BLM was retained in liposomes even after incubation at acidic pH for 36 h [47].

**Figure 1:**
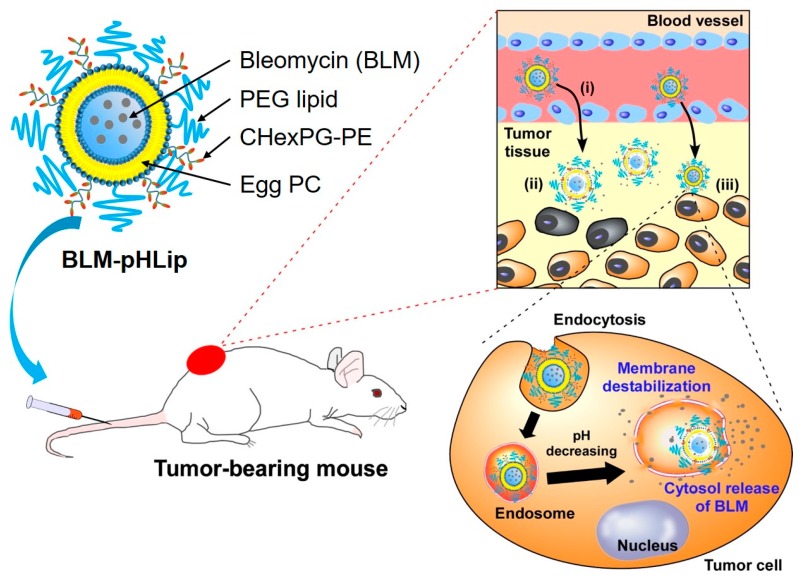
Design of bleomycin (BLM)-loaded pH-sensitive liposomes modified with poly(ethylene glycol) for intracellular drug delivery. (**i**) Accumulation of liposomes to tumor tissue via Enhanced Permeation and Retention (EPR) effect. (**ii**) BLM release responding to weakly acidic pH at tumor tissue. (**iii**) Some liposomes are taken up by tumor cells and release the drug in response to endosomal pH.

To improve the release behavior of BLM from liposomes and the therapeutic effect of BLM, BLM-loaded CHexPG-PE-incorporated EYPC liposomes were prepared. Then, their functions as a chemotherapeutic system were investigated (Figure 1). To provide colloidal stability in the bloodstream, PEG-lipids were also incorporated into the liposomes. After intravenous injection, these liposomes are expected to accumulate in the tumor tissue via EPR effect (Figure 1(i)), to release BLM responding to weakly acidic pH at tumor stroma (Figure 1(ii)), and to then be taken up by tumor cells. Subsequently, they will release BLM in response to acidic pH in endosomes or lysosomes (Figure 1(iii)). Here, the preparation of BLM-loaded CHexPG-PE liposomes with PEG-lipids was examined, along with their pH-responsive content release behavior, interaction with tumor cells, and in vitro/in vivo antitumor effects.

## 2. Materials and Methods

### 2.1. Materials

BLM (PubChem CID: 5360373; Bleo) was obtained from Nippon Kayaku (Tokyo, Japan). Egg yolk phosphatidylcholine (EYPC), *N*-[methoxy (polyethyleneglycol) 2000]-distearoyl phosphatidylethanolamine (PEG-PE), and CHexPG-PE, which is distearoyl phosphatidylethanolamine with 2-carboxycyclohexane-1-carboxylated poly(glycidol) groups, were obtained from NOF Co. (Tokyo, Japan). Pyranine and Triton X-100 were obtained from Tokyo Chemical Industries Ltd. (Tokyo, Japan). *p*-Xylene-bis-pyridinium bromide (DPX) was obtained from Sigma (St. Louis, MO, USA). 1,1′-Dioctadecyl-3,3,3′,3′-tetramethylindocarbocyanine perchlorate (DiI) was obtained from ThermoFisher (Waltham, MA, USA).

### 2.2. Cell Culture

Colon-26 cells (murine colon cancer cells; RIKEN cell bank, Tsukuba, Japan) were maintained as an adherent monolayer culture in RPMI 1640 medium (Invitrogen, Carlsbad, CA, USA) supplemented with 10% heat-inactivated fetal bovine serum (FBS, Nichirei Biosciences Inc., Tokyo, Japan) and PSN (5 mg/mL penicillin, 5 mg/mL streptomycin, and 10 mg/mL neomycin; Invitrogen), and incubated in 5% CO_2_ at 37 °C.

The cells were harvested from near-confluent cultures with brief exposure to a solution containing 0.25% trypsin and 1 mM EDTA·4Na solution with phenol red (Invitrogen). Trypsinization was stopped using RPMI 1640 containing 10% FBS. The cells were centrifuged and re-suspended in RPMI 1640. Trypan blue staining was used to assess cell viability.

### 2.3. Preparation of Liposomes 

Given amounts of EYPC (23.3 mg) and PEG-PE (3.74 mg) dissolved in chloroform and of CHexPG-PE (2.66 mg) dissolved in methanol were taken in a 10 mL round-bottom flask. Then, organic solvent was removed using a rotary evaporator and subsequent incubation for 3 h under vacuum to form a lipid thin film. The mixed thin membrane of EYPC, PEG-PE, and CHexPG-PE was dispersed in 2.5 mL of BLM solution (in Dulbecco’s phosphate buffered saline: PBS, 2 mg/mL). The liposome suspension was further hydrated by freezing and thawing, and was extruded through a polycarbonate membrane with a pore size of 100 nm. Free BLM was removed from the liposomes by repeated ultracentrifugation (55,000 rpm) for 1.5 h at 4 °C. The encapsulation efficiency of BLM in the liposomes was estimated from the absorbance of BLM at 242 nm for the BLM-loaded liposomes after addition of Triton X-100 (final concentration: 0.05%) using a UV–Vis spectrometer (Jasco V-560, Tokyo, Japan). The lipid-derived absorbance was corrected using the same liposomes without BLM. The encapsulation efficacy was 42.5 ± 6.6%. For cellular association, liposomes containing DiI were prepared as described above except that a lipid mixture containing DiI (0.1 mol %) was dispersed in the BLM solution.

For the encapsulation of pyranine, the dry membrane of a lipid mixture was dispersed in aqueous 35 mM pyranine, 50 mM DPX, and 25 mM phosphate solution (pH 8.8, 1.0 mL). The liposome suspension was further hydrated by freezing and thawing, and was extruded through a polycarbonate membrane with a pore size of 100 nm. The liposome suspension was purified using a Sepharose 4B column with PBS (pH 8.4). The typical lipid concentration of obtained liposomes was 5.0 × 10^−3^ M.

### 2.4. Dynamic Light Scattering and Zeta Potential

Diameters in PBS and zeta potentials in 0.1 mM phosphate aqueous solution of the liposomes (0.1 mM lipids) were measured using a Zetasizer Nano ZS ZEN3600 (Malvern Instruments Ltd., Worcestershire, UK). Data was obtained as an average of three measurements on different samples.

### 2.5. Release of Pyranine from Liposomes

Release of pyranine from the liposomes was measured as previously reported [31,32,33,34,35]. Liposomes encapsulating pyranine were added to PBS solutions of varying pH at 37 °C (final lipid concentration: 2.0 × 10^−5^ M) and fluorescence intensity (512 nm) of the mixed suspension was followed with excitation at 416 nm using a spectrofluorometer (Jasco FP-6500, Tokyo, Japan). The percent release of pyranine from liposomes was defined as

Release (%) = (*F*_t_ − *F*_i_)/(*F*_f_ − *F*_i_) × 100

where *F*_i_ and *F*_t_ denote the initial and intermediary fluorescence intensities of the liposome suspension, respectively. *F*_f_ is the fluorescence intensity of the liposome suspension after the addition of TritonX-100 (final concentration: 0.1%).

### 2.6. Cellular Association of Liposomes

Colon-26 cells (1.0 × 10^5^ cells) cultured overnight in 12-well plates were washed twice with PBS, and then incubated in 10% FBS-containing RPMI 1640 medium (0.5 mL). The DiI-labeled liposomes (1 mM lipid concentration, 0.5 mL) were gently added to the cells and incubated for 4 h at 37 °C. After incubation, the cells were washed three times with PBS. The fluorescence intensity of these cells was determined by flow cytometric analysis (CytoFlex, Beckman Coulter, Inc., Brea, CA, USA). The cellular autofluorescence was subtracted from the data.

### 2.7. Cellular Cytotoxicity of Liposomes

Colon-26 cells (1.0 × 10^4^ cells) cultured overnight in 96-well plates were washed twice with PBS, and then incubated with free BLM or pH-sensitive liposomes with or without BLM at various concentrations for 24 h at 37 °C. After washing by PBS, the cell viability of the Colon-26 cells was assessed with Cell Count Reagent SF (nacalai tesque, Kyoto, Japan) according to the manufacturer’s instructions. The absorbance (optical density, OD) was measured at 450 nm using a Microplate Reader (SH-8000, CORONA ELECTRIC Co., Ltd., Ibaraki, Japan). Each group included three replicates. 

### 2.8. Ethics Statement

Animal use and procedures were approved by the Animal Research Committee of Tottori University (project number: 13-T-43). The study is in accordance with the Institute of Laboratory Animal Resources guidelines for the use of experimental animals. 

### 2.9. Animals and Preparation of Tumor-Bearing Mice Model

Six-week-old, female BALB/c mice (CLEA Japan, Tokyo, Japan) were maintained under conventional conditions and provided with a standard pellet diet and water ad libitum. Using a 26-gauge syringe, Colon-26 cells were inoculated subcutaneously in the shaved lower dorsum of mice at a concentration of 1.0 × 10^6^ cells/0.1 mL per mouse.

### 2.10. Therapeutic Effects of Liposomes on Tumor-Bearing Mice

Tumor-bearing mice were allocated randomly to six groups of six animals each: (1) control group; (2) BLM (10 mg/kg); (3) BLM-loaded PEG-modified liposomes (BLM-Lipo 10 mg/kg); (4) BLM-loaded CHexPG-PE/PEG-modified liposomes (BLM-pHLipo, 1 mg/kg); (5) BLM-loaded CHexPG-PE/PEG-modified liposomes (BLM-pHLipo, 5 mg/kg); and (6) BLM-loaded CHexPG-PE/PEG-modified liposomes (BLM-pHLipo, 10 mg/kg). Mice were intravenously injected via tail with 200 μL of BLM (10 mg/kg), BLM-Lipo (10 mg/kg), and BLM-pHLipo (1, 5, and 10 mg/kg) under anesthesia with isoflurane (Pfizer, Tokyo, Japan). The body weight and tumor volume of mice were measured every other day. Subcutaneous tumor dimensions were determined using calipers. Tumor volume was calculated using the formula length × width^2^ × 0.5. The experiment was terminated on day 12. All mice were sacrificed by cervical dislocation, and the tumors, spleens, livers, and lungs were harvested.

### 2.11. Histologic Analysis

The specimens were fixed in 4% buffered formalin, embedded in paraffin, sectioned to a thickness of 4 μm, stained with hematoxylin and eosin, and observed using optical microscopy (Olympus BX51, Tokyo, Japan).

### 2.12. Statistical Analysis

Data was analyzed using the Friedman test. A *p* value < 0.05 was considered statistically significant. Statistical analyses were performed using GraphPad Prism version 6 (GraphPad Software Inc., La Jolla, CA, USA). 

## 3. Results and Discussion

### 3.1. Characterization of Liposomes

**Figure 2:**
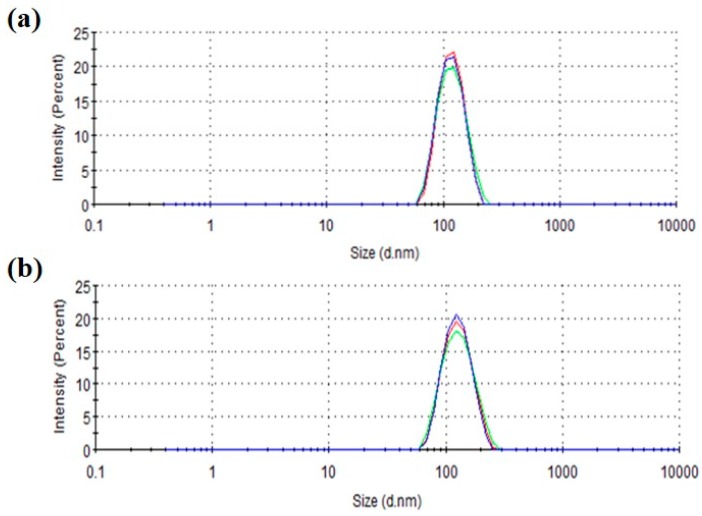
Intensity-weighted size distribution of BLM-loaded PEG liposomes without ((**a**) BLM-Lipo) and with CHexPG-PE ((**b**) BLM-pHLipo). Red, blue and green lines indicate the results for three different measurements.

Bleomycin (BLM)-loaded liposomes were prepared using the lipid film hydration method. The encapsulation efficiency of BLM in liposomes was determined as 42.5 ± 6.6% using spectroscopic measurements. Dynamic light scattering analysis of the liposomes revealed the quite narrow size distribution of obtained liposomes with a size of around 115 nm, which almost corresponds to the pore size of the polycarbonate membrane during extrusion and is a suitable size for EPR effect (Table 1 and Figure 2). Highly hydrated PEG molecules with high mobility on the liposome surface might suppress the collision between liposomes and provide high colloidal stability to these liposomes, as reported previously in the literature [48,49,50]. Compared with liposomes without CHexPG-PE (BLM-Lipo), liposomes containing CHexPG-PE (BLM-pHLipo) showed low and negative zeta potentials (Table 1). This result directly reflects the surface modification of CHexPG-PE-including carboxyl groups.

### 3.2. pH-Sensitivity of Liposomes

**Figure 3:**
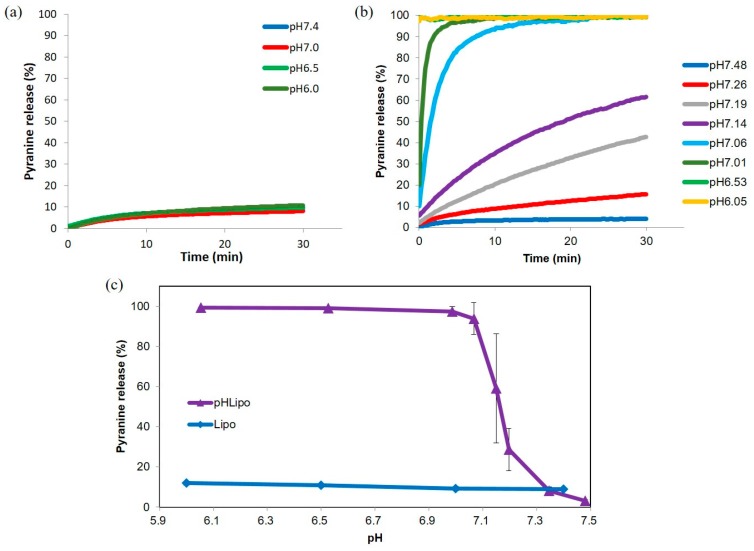
Typical time courses (**a**,**b**) and pH-dependence after 30 min incubation (**c**) of pyranine release from PEG liposomes modified with or without CHexPG-PE at 37 °C.

Incorporation of CHexPG-PE into EYPC liposomes provided pH-sensitive properties to the parent liposomes [35]. In this study, PEG-PE was further incorporated to provide colloidal stability and long-term circulation properties to the liposomes. However, a PEG chain might interfere with the pH-sensitive properties of CHexPG-PE. Therefore, the pH-responsive content release behaviors of PEG-modified liposomes were examined (Figure 3). A fluorescent dye (pyranine) and its quencher (DPX) were encapsulated into the liposomes. When both pyranine and DPX are encapsulated into liposomes, the pyranine fluorescence is quenched by DPX. In contrast, when pyranine and DPX molecules are released from liposomes after destabilization of the liposomal membrane, pyranine fluorescence increases significantly [51]. Using this phenomenon, the pyranine release behavior from the liposomes was investigated. In the case of the PEG-modified EYPC liposomes without CHexPG-PE, the release of pyranine was less than 10%, irrespective of the environmental pH (Figure 3a), which indicates that liposomes without CHexPG-PE stably retained pyranine molecules. Liposomes with CHexPG-PE showed no content release at pH 7.5 or 7.3, although marked pyranine release was observed from liposomes at pH less than 7.2 (Figure 3b). At pH 7.0, all pyranine molecules were released from liposomes within 5 min. According to the result of acid–base titration for CHexPG without the PE moiety, the protonation of carboxyl groups in CHexPG was promoted remarkably at around pH 7.0, which corresponds to the p*K*_a_ value of CHexPG (Figure S2). After protonation of carboxyl groups in CHexPG-PE on the liposomal surface, polymer chains might possess hydrophobic characteristics and take shrinking globular conformation because of the cancellation of electrostatic repulsion between carboxylates and hydrophobic interaction between bulky cyclohexyl groups [52]. Hydrophobized CHexPG chains might be inserted into the liposomal membrane, which causes a decrease of bilayer lipid packing and destabilization of the liposomal membrane, leading to pyranine release from the liposomes. Figure 3c also shows the sharp pH-responsiveness of CHexPG-PE-incorporated liposomes, which is almost identical to previous results obtained for CHexPG-PE liposomes without PEG-PE [35]. Therefore, incorporation of PEG-PE might affect the pH-sensitivity of CHexPG-PE only slightly.

### 3.3. Cellular Association of Liposomes

**Figure 4:**
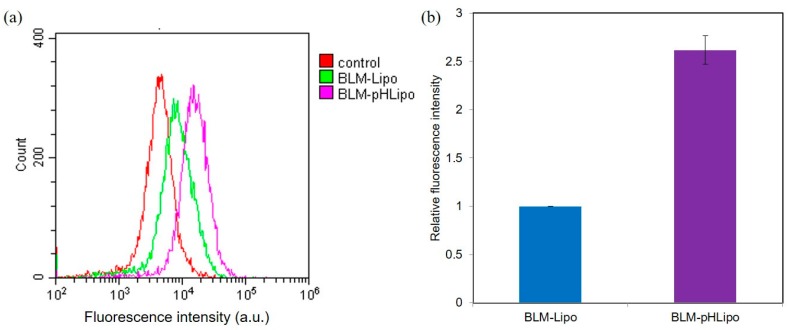
Fluorescence distribution (**a**) and relative fluorescence intensity (**b**) for Colon-26 cells treated with fluorescence dye(DiI)-labeled BLM-Lipo or BLM-pHLipo for 4 h at 37 °C. Lipid concentration was 0.5 mM.

Next, the interaction of liposomes with cells was investigated. Fluorescently labeled liposomes were applied to Colon-26 cells for 4 h. Then, cellular fluorescence was detected using a flow cytometer (Figure 4). The distribution of cellular fluorescence shifted to the higher fluorescence region under liposome treatments compared with cellular autofluorescence alone (Figure 4a), indicating efficient cellular association of DiI-labeled liposomes. Incorporation of CHexPG-PE into the liposomes decreased the zeta potential (Table 1), which might suppress cellular association because of electrostatic repulsion with the anionic cell surface. Furthermore, cellular association experiments were conducted in the presence of serum. The zeta potential of CHexPG-PE-incorporated PEG liposomes in the presence of serum was −18.0 ± 5.5 mV, which is a slightly higher value compared with the case in the absence of serum (Table 1). Although serum proteins might associate onto the liposome surface and slightly shield the negative charge derived from CHexPG-PE, the liposomes still possessed negative charge. However, CHexPG-PE-incorporated PEG liposomes showed 2.5 times higher cellular association than the PEG liposomes without CHexPG-PE (Figure 4b). This higher cellular association might be attributed to hydrophobic interaction derived from bulky cyclohexyl units with cells as previously reported in the literature [35]. Therefore, CHexPG-PE-incorporated PEG liposomes associated efficiently to cancer cells, even in the presence of many proteins.

### 3.4. Cellular Cytotoxicity of Liposomes

**Figure 5:**
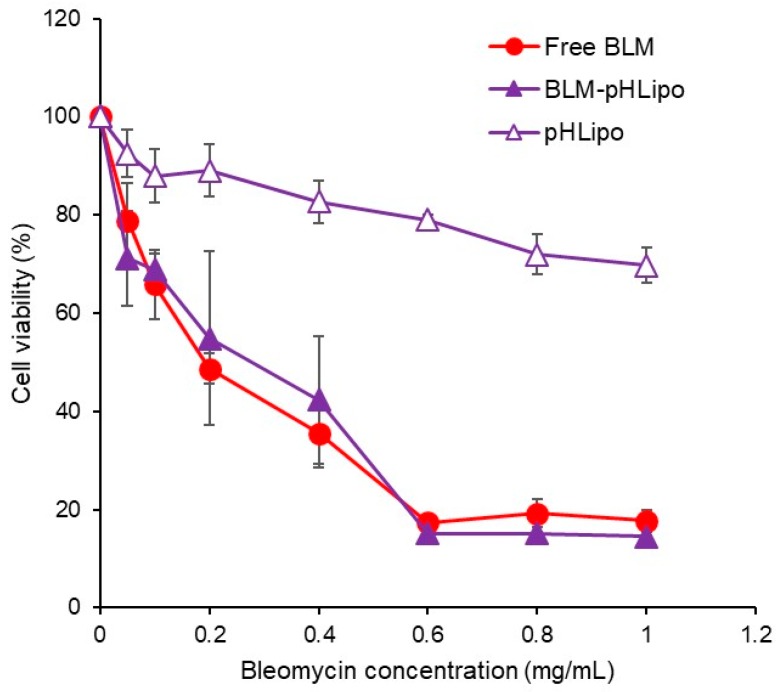
Effect of BLM-loaded pH-sensitive liposomes on cell viability. Colon-26 cells were treated with free BLM, pHLipo, or BLM-pHLipo at varying BLM concentrations for 24 h. The cell viability was evaluated. Each plot shows the mean ± SD (*n* = 3).

Considering the delivery performance of CHexPG-PE-incorporated PEG liposomes, BLM delivery by these liposomes and their cytotoxicity to cancer cells were examined (Figure 5). Colon-26 cells were treated with free BLM or BLM-loaded CHexPG-PE-incorporated PEG liposomes for 24 h. Cell viability was then measured. As shown in Figure 5, BLM-loaded liposomes exhibited identical cytotoxicity to Colon-26 cells to that achieved by free BLM. This result might reflect the efficient cellular association of liposomes to Colon-26 cells (Figure 4). In addition, the same liposomes without BLM (pHLipo) showed only slight cellular toxicity under experimental conditions. BLM-loaded CHexPG-PE-incorporated PEG liposomes might release BLM molecules responding to weakly acidic pH in early endosomes after the internalization of liposomes, and the released BLM molecules then interact with DNA in the cells, leading to cell death.

### 3.5. Therapeutic Effect of Liposomes on Tumor-Bearing Mice

**Figure 6:**
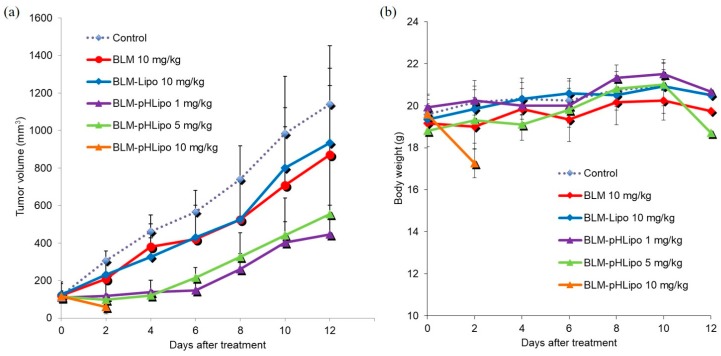
In vivo antitumor effect of BLM-loaded liposomes. Free BLM, BLM-Lipo, or BLM-pHLipo was administered intravenously to Colon-26 tumor-bearing BALB/c mice. Changes in (**a**) tumor volume (mm^3^) and (**b**) body weight were monitored (*n* = 6). Control vs. BLM-pHLipo (1 mg/kg): *p* = 0.001; Control vs. BLM-pHLipo (5 mg/kg): *p* = 0.023; BLM-Lipo (10 mg/kg) vs. BLM-pHLipo (1 mg/kg): *p* = 0.013; Friedman test.

Next, the in vivo therapeutic effects of the liposomes were examined. Free BLM or BLM-loaded liposomes were administered intravenously to Colon-26-bearing mice. As shown in Figure 6a, free BLM exhibited slight suppression of tumor growth compared with the control group, whereas BLM-loaded CHexPG-PE-incorporated PEG liposomes strongly suppressed tumor growth until day 6 even though their BLM dosages were lower than those for free BLM. In addition, BLM-loaded pH-insensitive liposomes (BLM-Lipo) induced almost identical antitumor effects to those of free BLM. These results suggest that BLM-loaded CHexPG-PE-incorporated PEG liposomes delivered BLM to tumor tissues efficiently via the EPR effect and released BLM at the tumor site or inside of tumor cells, responding to weakly acidic pH at the tumor stroma or early endosomes of tumor cells. However, when BLM-loaded CHexPG-PE-incorporated PEG liposomes were administered at a high dosage (10 mg/kg), the mice died on day 4, although tumors in these mice disappeared completely. Reflecting these results, CHexPG-PE-incorporated PEG liposomes with a high dosage (10 mg/kg) showed a remarkable decrease of the mice body weight after treatment, whereas other treatment groups only slightly affected the mice body weight (Figure 6b). These results indicate that high doses of BLM-loaded pH-sensitive liposomes might release BLM not only at tumor tissues but also at other normal organs, thereby causing severe adverse effects to mice, as described in the next histological analysis. Among the examined dosages for CHexPG-PE-incorporated PEG liposomes, 1 mg/kg showed the strongest therapeutic effects. After 6 days, tumor reoccurrence was observed for CHexPG-PE-incorporated PEG liposomes, which indicates that administered liposomes and BLM might be excluded from mice within 6 days. Therefore, once-weekly treatment of BLM-loaded pH-sensitive liposomes is expected to achieve complete tumor growth suppression.

### 3.6. Histological Analysis

**Figure 7:**
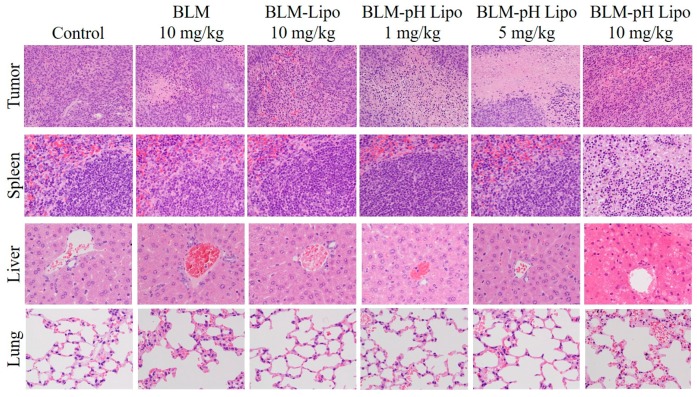
Evaluation of systemic toxicities by hematoxylin and eosin (H&E) staining showing histopathological changes in tumor, spleen, liver, and lung. In the cases of the control, BLM, BLM-Lipo (10 mg/kg), BLM-pHLipo (1 mg/kg) and BLM-pHLipo (5 mg/kg) groups, organs were isolated 12 days after treatment. In the case of BLM-pHLipo (10 mg/kg) groups, organs were isolated 4 days after treatment. Tumors (×20). Spleen, liver, and lung (×40).

Next, a histological analysis of mice treated with BLM or liposomes was conducted to elucidate the mechanisms of therapeutic effects by low-dosage pH-sensitive liposomes and adverse effects by high dosage pH-sensitive liposomes. On day 12, the tumor, spleen, liver, and lungs were excised from the mice. Each was sectioned to 4 μm thickness; then, hematoxylin and eosin (H&E) staining was performed (Figure 7). In the case of mice treated with high-dosage pH-sensitive liposomes, organs were collected on Day 4. Compared with the control group, necrotic areas in which cells lost nuclei or had deformed nuclei were observed in free BLM. In the case of BLM-loaded pH-insensitive liposomes, necrotic areas were observed around vessels. In contrast, most parts of tumor tissues underwent necrosis for the cases of BLM-loaded pH-sensitive liposomes (1 and 5 mg/kg), which correlates to the strong antitumor effects of these liposomes (Figure 6). In the case of BLM-loaded pH-sensitive liposomes at a high dosage (10 mg/kg), tumor tissue was necrotic. Furthermore, severe necrosis and degenerated lymph follicles were observed in the spleen and severe necrosis of liver parenchyma around the portal triad were observed in the liver (Figure 7). In other groups, remarkable change was rarely observed in the spleen and liver. In lung tissues, alveolar epithelial cells were moderately thickened after treatment with free BLM and BLM-loaded pH-sensitive liposomes (5 and 10 mg/kg). These results suggest that, at the high dosage, BLM-loaded pH-sensitive liposomes were trapped in mononuclear phagocyte systems (MPS) in the spleen, liver, and lungs and released BLM at these tissues, leading to severe toxicity to mice. By contrast, the same dosage of pH-insensitive liposomes showed only slight toxicity to mice (Figure 6b and Figure 7). Phagocytic cells, such as macrophages, are known to have scavenger receptors that recognize anionic molecules such as phosphatidylserine on apoptotic cells and eliminate these cells [53,54]. Carboxylated polymer-modified liposomes were also taken up efficiently by macrophages or dendritic cells via recognition by scavenger receptors [33,55,56]. Considering that CHexPG-PE-incorporated liposomes possess negative charge derived from carboxyl groups in the CHexPG moiety (Table 1), it is likely that these liposomes are recognized by phagocytic cells even in the presence of hydrated PEG chains on the liposome surface, leading to the trapping of liposomes in the spleen, liver, and lungs. For low-dosage BLM-loaded pH-sensitive liposomes, a portion of the liposomes might be taken up by MPS, but the amounts of BLM in major organs might be lower than toxic levels. Therefore, escape from MPS recognition and selective accumulation to tumors would further improve the therapeutic effects of BLM-loaded pH-sensitive liposomes and reduce the toxicity to mice.

### 3.7. Effect of PEG Length on Therapeutic Effect

To reduce recognition by MPS, PEG length on the liposomes was changed by using PEG5000-PE for liposome preparation instead of PEG2000-PE. BLM-loaded pH-sensitive liposomes containing PEG5000-PE were injected intravenously into tumor-bearing mice. The liposome antitumor effects and mouse body weights were monitored (Figure S3), and histological evaluation on Day 12 was also performed (Figure S4). In the case of low-dosage liposomes (1 mg/kg), the tumor growth pattern was almost identical to that of the control groups (Figure S3a), but necrotic areas were observed in the tumor section (Figure S4). At high dosages (5 or 10 mg/kg), tumor growth was suppressed for a duration of 4 days (Figure S3a) reflecting the necrosis in tumor sections (Figure S4). Importantly, a remarkable decrease of body weight only slightly occurred (Figure S3b). No severe toxicity was observed in the spleen or liver (Figure S4), unlike the cases with the PEG2000-PE-based liposomes. These results suggest that an increase in PEG length avoided recognition by phagocytic cells in the liver and spleen. However, alveolar epithelial cells thickened significantly with increasing BLM dosage (Figure S4). PEG5000-liposomes might accumulate not only in tumors, but also in lungs. Aggregation of liposomes during blood circulation might cause entrapment of liposomes into blood capillaries in the lung. However, intrinsic sizes of PEG2000-PE-based liposomes and PEG5000-PE-based liposomes were hardly affected in the presence of serum (Figure S5). Therefore, other differences of PEG5000-PE-based liposomes in terms of physicochemical properties or morphological change under shear stress in blood stream might induce lung accumulation compared with PEG2000-PE-based liposomes. Further investigations, such as evaluation of liposomal biodistribution, are required to elucidate the mechanism of this unexpected lung accumulation.

In this study, the antitumor effects of BLM-loaded liposomes were achieved by a single injection. Large doses of BLM-loaded pH-sensitive liposomes tend to show not only a therapeutic effect but also severe adverse effects. Instead, repeated injection of low-dosage BLM-loaded liposomes is expected to improve their therapeutic effect and to reduce toxicity to mice. In addition, the selection of suitable anticancer drugs or combination with other bioactive molecules is also expected to improve their antitumor effects. For example, combination with an immunosuppressive reagent (tacrolimus) is expected to suppress lung fibrosis induced by BLM accumulation in lungs [57]. Further investigations must be undertaken for the establishment of efficient cancer chemotherapeutic systems using pH-sensitive polymer–lipid-incorporated liposomes.

## 4. Conclusions

This study investigated the DDS function of pH-sensitive polymer–lipid- and PEG-PE-incorporated liposomes encapsulating BLM as an anticancer drug. These liposomes showed content release responding to a pH decrease and were taken up by cancer cells efficiently, leading to strong in vitro cytotoxicity. Intravenous administration of these liposomes strongly suppressed tumor growth. However, high doses of BLM using pH-sensitive polymer–lipid-incorporated liposomes showed severe toxicity to mice. An increase of PEG length on the liposome surface decreased the toxicity to the liver and spleen, whereas lung toxicity still remained. Further improvements such as optimization of PEG density or lipid compositions to avoid recognition by MPS, and the introduction of tumor-selective ligands to provide selectivity to tumor tissues, are still needed to achieve an efficient chemotherapeutic system with lessened adverse effects.

## Figures and Tables

**Table 1:** Characterization of liposomes. BLM-Lipo: BLM-loaded PEG-modified liposomes, BLM-pHLipo: BLM-loaded CHexPG-PE/PEG-modified liposomes, PDI: polydispersity index.

Liposome	Size (nm)	PDI	Zeta Potential (mV)
BLM-Lipo	115.3 ± 3.0	0.10 ± 0.03	−19.2 ± 5.0
BLM-pHLipo	115.5 ± 4.2	0.06 ± 0.01	−29.7 ± 5.4

## Supplementary Materials

The following are available online at www.mdpi.com/2073-4360/10/1/74/s1. Figure S1: Structures of CHexPG-PE and BLM, Figure S2: Titration curve of CHexPG, Figure S3: In vivo antitumor effect using PEG5000-PE liposomes, and Figure S4: Histological analysis for Figure S3, Figure S5: Effect of serum on the size distribution of liposomes.
