# Supplementary material for: Bleomycin-Loaded pH-Sensitive Polymer–Lipid-Incorporated Liposomes for Cancer Chemotherapy
Source: Polymers (Basel). 2018 Jan 15;10(1):74. doi: 10.3390/polym10010074 (PMC6415073; doi:10.3390/polym10010074)
Supplement: Supplementary file 1 [file polymers-10-00074-s001.docx]

**Supplementary Materials**

Bleomycin-loaded pH-sensitive polymer-lipid-incorporated liposomes for cancer chemotherapy

Eiji Yuba ^1,^*, Tomohiro Osaki ^2,^*, Misato Ono ^2^, Shinjae Park ^1^, Atsushi Harada ^1^, Masamichi Yamashita ^2^, Kazuo Azuma ^2^, Takeshi Tsuka ^2^, Norihiko Ito ^2^, Tomohiro Imagawa ^2^ and Yoshiharu Okamoto ^2^

^1^ Department of Applied Chemistry, Graduate School of Engineering, Osaka Prefecture University, 1–1 Gakuen–cho, Naka–ku, Sakai, Osaka 599-8531, Japan; ma105114@edu.osakafu-u.ac.jp (S.P.); harada@chem.osakafu-u.ac.jp (A.H.)

^2^ Joint Department of Veterinary Clinical Medicine, Faculty of Agriculture, Tottori University, 4–101 Koyama–Minami, Tottori 680-8553, Japan; misatosu202@gmail.com(M.O.); kazu-azuma@muses.tottori-u.ac.jp (K.A.); ymurahata@muses.tottori-u.ac.jp (Y.M.); tsuka@muses.tottori-u.ac.jp (T.T.); taromobile@me.com (N.I.); imagawat@muses.tottori-u.ac.jp (T.I.); yokamoto@muses.tottori-u.ac.jp (Y.O.)

***** Correspondence: yuba@chem.osakafu-u.ac.jp; Tel.: +81-72-254-9913; Fax: +81-72-254-9330 (E.Y.),

tosaki@muses.tottori-u.ac.jp; Tel&Fax: +81-857-31-5434 (T.O.)


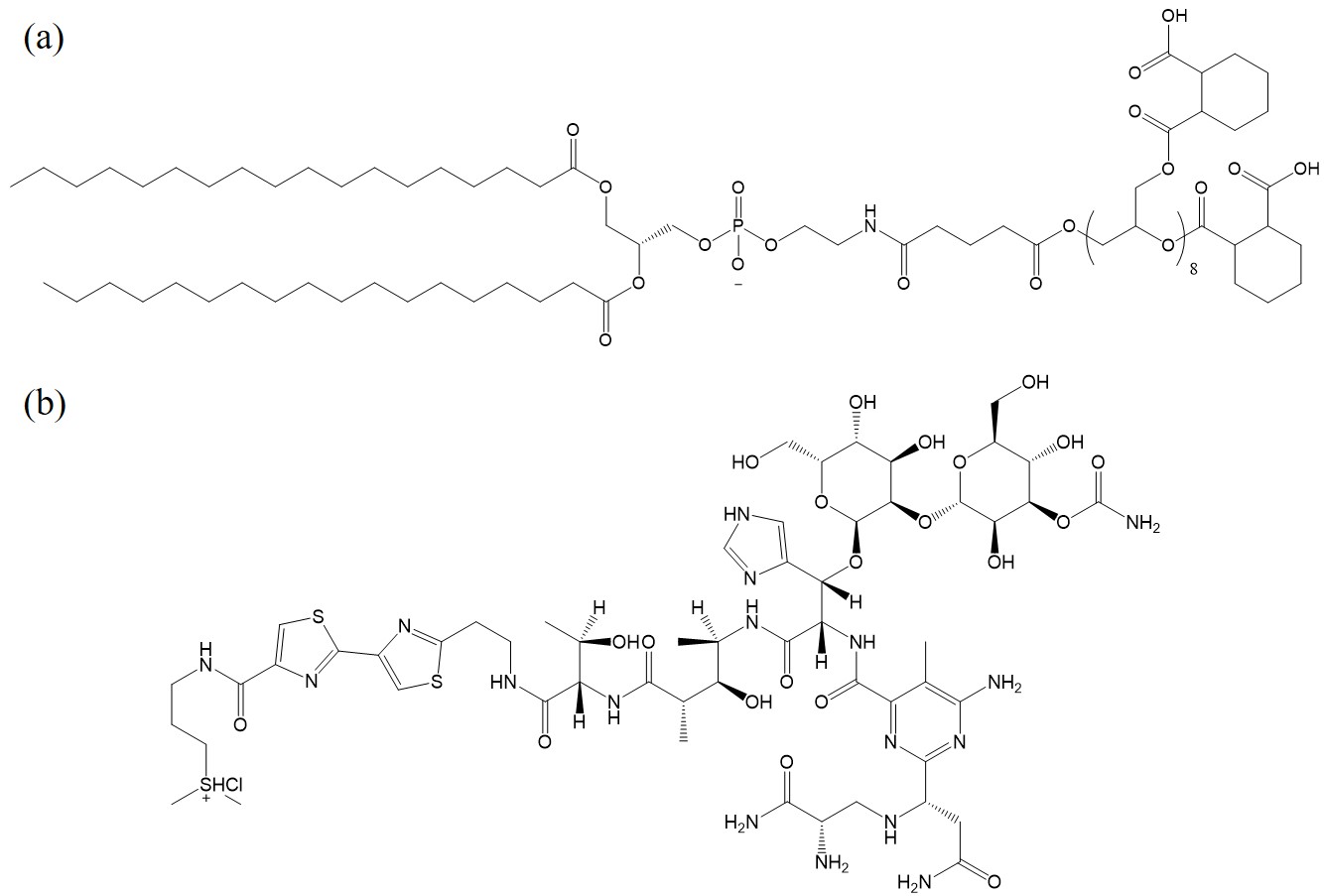


**Figure S1.** Chemical structures of CHexPG-PE (a) and bleomycin (b).


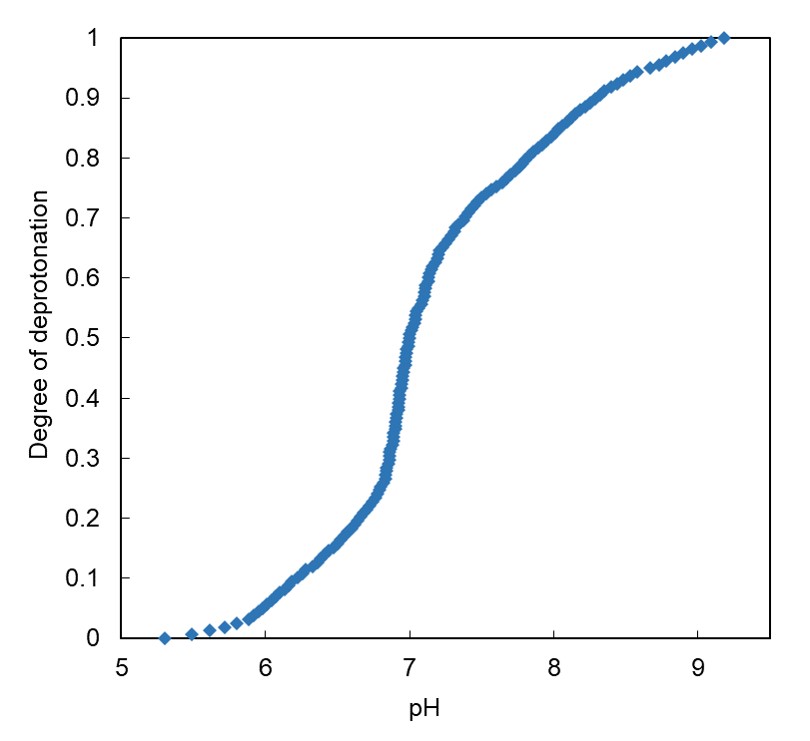


**Figure S2.** Titration curve of CHexPG. CHexPG with 91% of CHex units was synthesized according to previous literature (*Bioconjugate Chemistrty*, **19**, 1040-1048 (2008)) and was used for acid-base titration. p*K*a: 7.0.


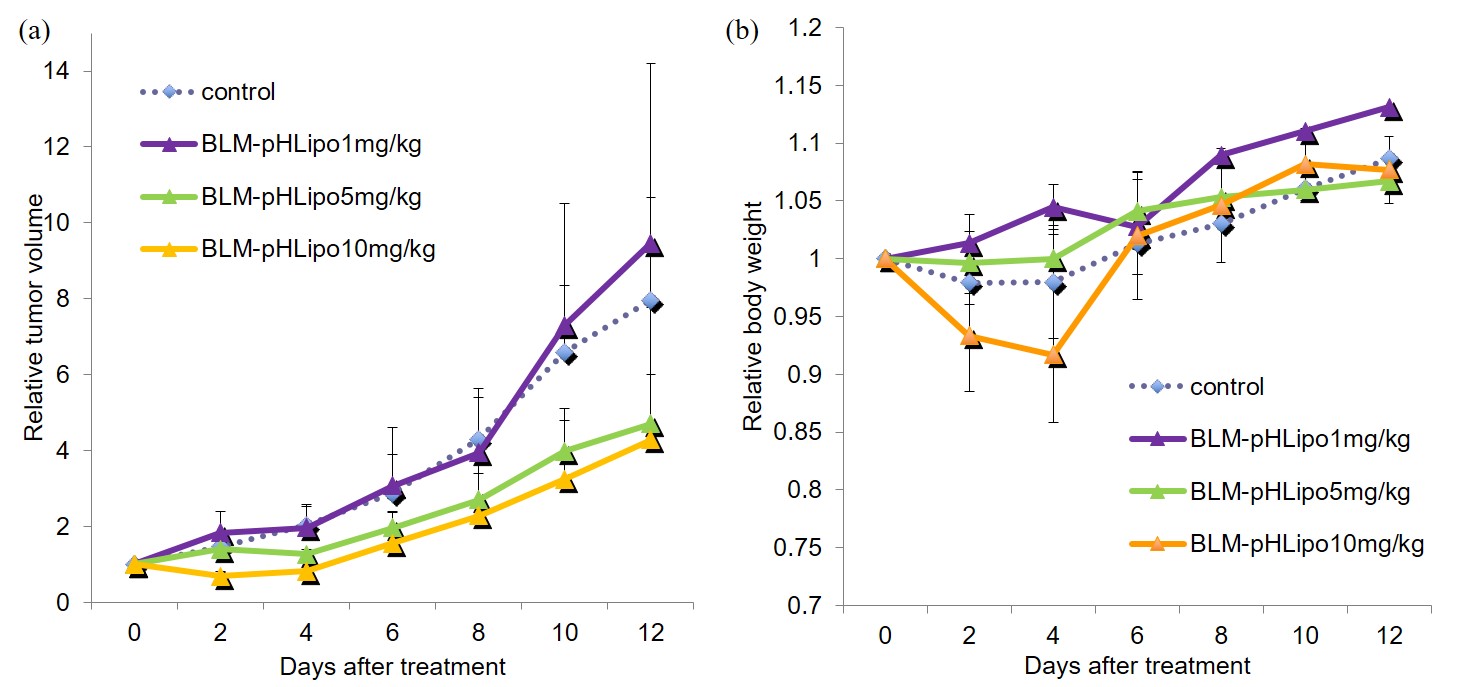


**Figure S3.** *In vivo* antitumor effect of BLM-loaded liposomes with PEG5000-DSPE. BLM-pHLipo (BLM: 1, 5, 10 mg/kg) were intravenously administered to Colon-26 tumor-bearing BALB/c mice. Change in (a) relative tumor volume and (b) relative body weight were monitored (n=6).


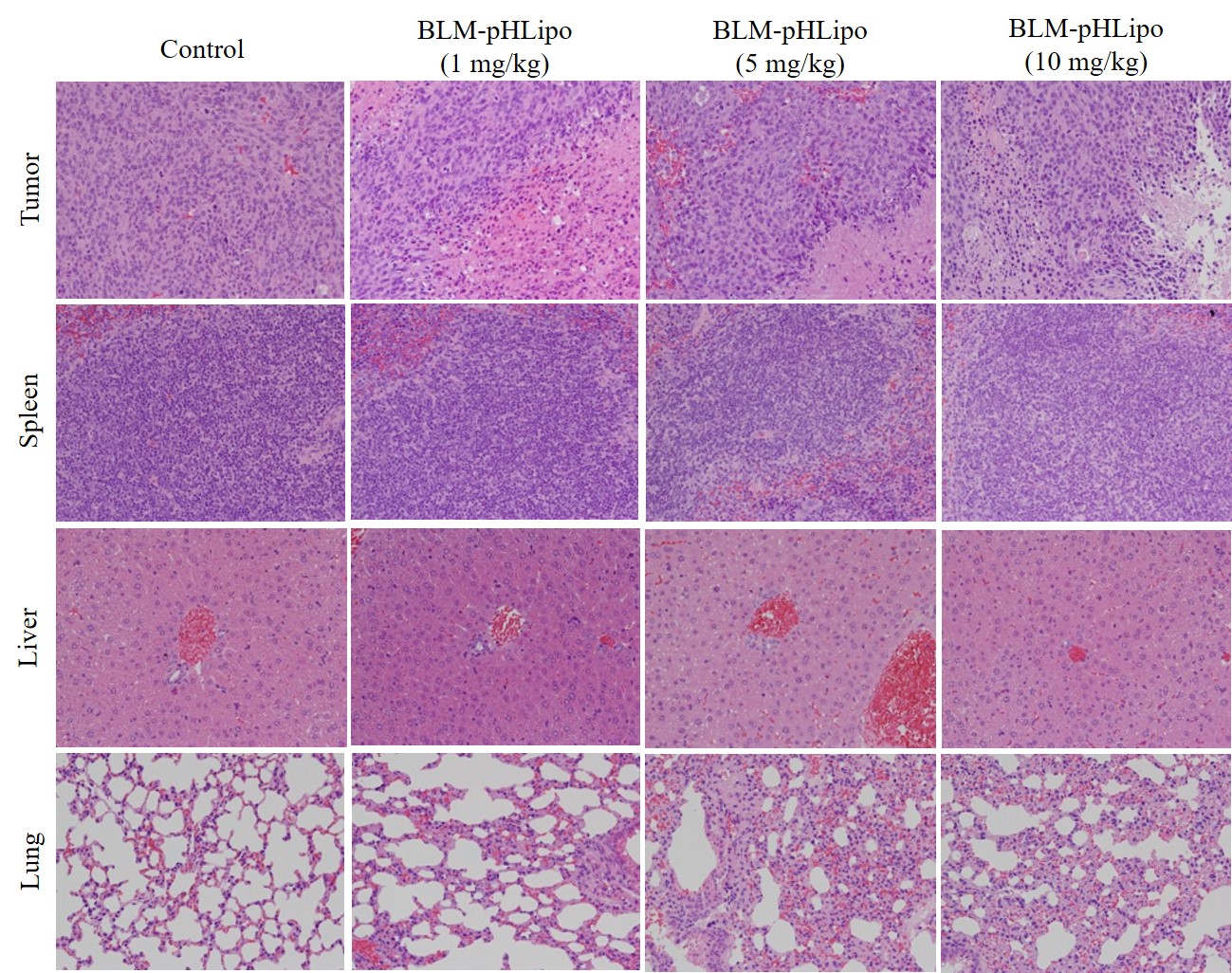


**Figure S4.** Evaluation of systemic toxicities by H&E staining showing histopathological changes in tumor, spleen, liver, and lung isolated 12 days after treatment in Figure S3. Tumors (×20). Spleen, liver, and lung (×40).


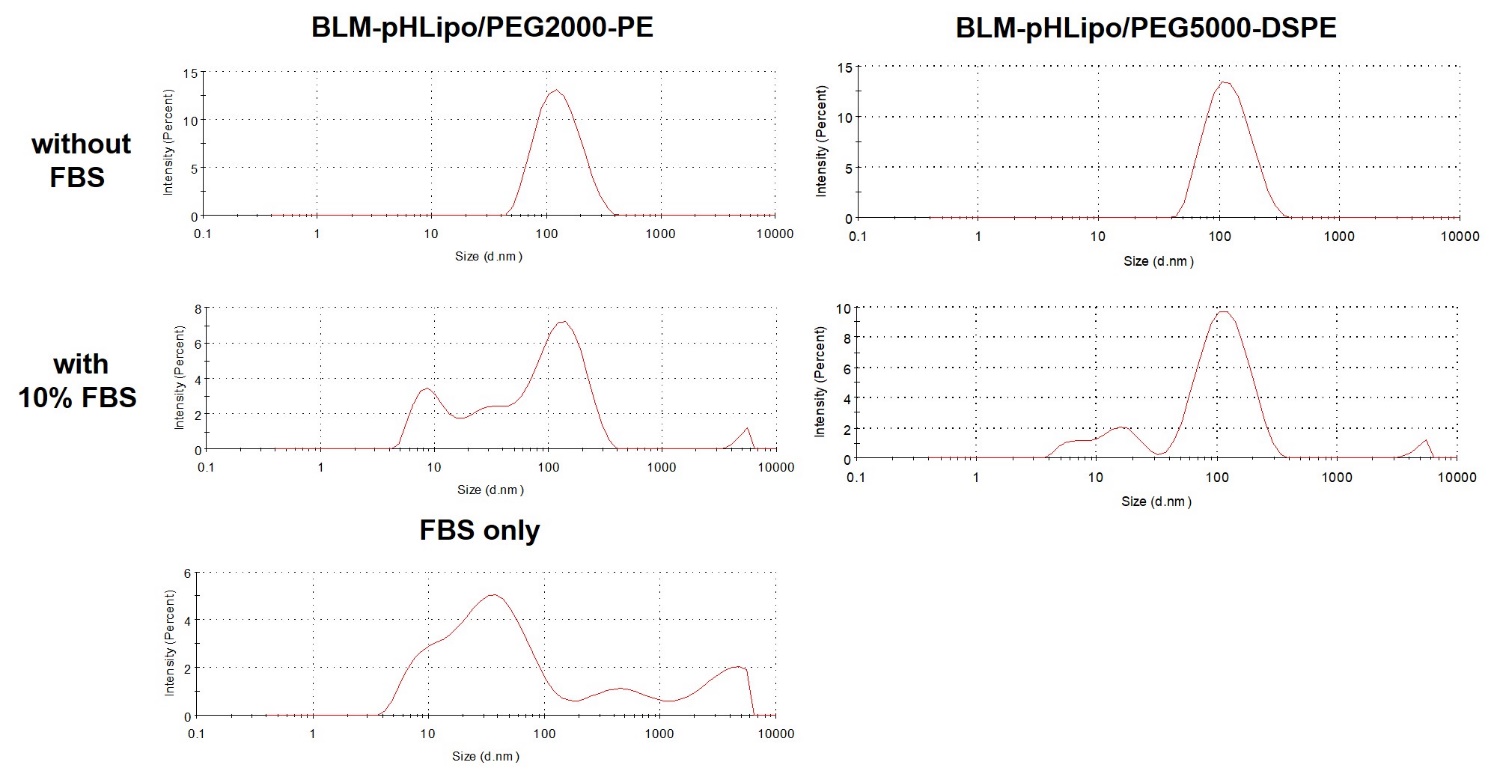


**Figure S5.** Intensity-weighted size distribution of BLM-loaded CHexPG-PE liposomes containing PEG2000-PE or PEG5000-PE in the presence or absence of 10% FBS. DLS result for FBS was also shown.
